# Mutation detection in Chinese patients with familial hypercholesterolemia

**DOI:** 10.1186/s40064-016-3763-3

**Published:** 2016-12-12

**Authors:** Ran Du, Liang-Liang Fan, Min-Jie Lin, Zhi-Jian He, Hao Huang, Ya-Qin Chen, Jing-Jing Li, Kun Xia, Shui-Ping Zhao, Rong Xiang

**Affiliations:** 1 The State Key Laboratory of Medical Genetics, School of Life Sciences, Central South University, Changsha, 410013 China; 2 Department of Cardiology, The Second Xiangya Hospital of Central South University, Changsha, 410011 China

**Keywords:** Familial hypercholesterolemia, Mutation, *LDLR*

## Abstract

**Background:**

Familial hypercholesterolemia (FH) is the first molecularly and clinically characterized genetic disease of lipid metabolism. It is an autosomal dominant disorder with significantly elevated levels of total cholesterol and low density of lipoprotein cholesterol in serum, which would lead to extensive xanthomas and premature coronary heart disease. Mutations in *low density lipoprotein receptor* (*LDLR*), *proprotein convertase subtilisin/kexin type 9* and *Apo lipoprotein B*-*100* (*APOB*) have been identified to be the underlying cause of this disease.

**Methods:**

Genetic testing and reports of the mutations in the Chinese population are still limited. In this study, 11 unrelated Chinese FH families were enrolled to detect the candidate gene variants by DNA direct sequencing.

**Results and conclusion:**

We identified 12 mutations (11 in *LDLR* and one in *APOB*) in ten FH families. Three novel *LDLR* mutations (c.516C>A/p.D172E, c.1720C>A/p.R574S and c.760C>T/p.Q254X) were identified and co-segregated with the affected individuals in the families. Our discoveries not only further supports the significant role of LDLR in FH, but also expands the spectrum of *LDLR* mutations. These new insights will contribute to the genetic diagnosis and counseling of FH patients.

## Background

Dyslipidemia is a common disorder of lipid metabolism and major cardiovascular risk factor, accounting for 54% of population-attributable risk for myocardial infarction (Yusuf et al. 2004). Familial hypercholesterolemia (FH, OMIM#143890) is one of the most severe lipid dysfunctions, characterized by elevated total cholesterol and low density of lipoprotein cholesterol amounts in serum (Jannes et al. 2015). It is inherited in an autosomal dominant fashion, with frequencies of heterozygotes and homozygotes estimated at 1:200 and 1:300,000 worldwide (Foody and Vishwanath 2016). Total cholesterol and LDL-C concentrations in heterozygous patients often range between 9 and 14 mmol/L and 5–10 mmol/L, whereas homozygous patients show levels from 17 to 26 mmol/L and >10 mmol/L, respectively (European Association for Cardiovascular Prevention & Rehabilitation et al. 2011; Goldberg et al. 2011; Hovingh et al. 2013). Such high plasma TC and LDL-C levels may result in xanthelasmas and atherosclerotic plaques, the primary factors causing premature coronary heart disease (CHD) (Najam and Ray 2015). However, the levels of TC and LDL-C can be effectively reduced by statin (Vogt 2015).

To date, more than 1741 *low density lipoprotein*-*receptor gene* (*LDLR*) variants have been reported in the Human Gene Mutation Database (http://www.hgmd.cf.ac.uk/ac/index.php) (Lahtinen et al. 2015). Meanwhile, two distinct disease-causing genes were identified in FH patients: *proprotein convertase subtilisin/kexin type9* (*PCSK9*) (Al-Mashhadi et al. 2013) and *Apo lipoprotein B*-*100* (*APOB*) (Alves et al. 2014). The clinical phenotypes resulting from these gene mutations vary. For example, *APOB* mutations may cause the least severe phenotype of the three (Soutar and Naoumova 2007). Besides *LDLR*, *APOB* and *PCSK9* mutations, some copy number variants (CNVs) (Myocardial Infarction Genetics, Kathiresan et al. 2009; Costelloe et al. 2012) and rare mutations in associated genes, such as *LDLRAP1* (Maglio et al. 2014), *PNPLA5* (Lange et al. 2014) and *APOC3* (Jorgensen et al. 2014) have also been reported in FH patients.

*LDLR* gene mutations represent 85–90% of disease-causing mutations in FH patients (Futema et al. 2014), however, most countries (including China) do not have valid nationwide registries for FH. Indeed, no more than 20 studies have assessed Chinese FH patients using genetic analysis, and novel variants identified remain scarce (Dai et al. 2011).

Therefore, in this study we investigated the possible causative gene in Chinese FH families. We identified three novel mutations (c.516C>A/p.D172E, c.1720C>A/p.R574S and c.760C>T/p.Q254X) in the affected members of their families. Based on the best of our knowledge, these mutations have not been reported in previous studies and were not presented in either our control cohorts, dbSNP or Exome Variant Server database (http://evs.gs.washington.edu/EVS/).

## Methods

The Review Board of The Second Xiangya Hospital of the Central South University has approved this research. All related subjects have consented to this study.

### Patients and subjects

Eleven unrelated Chinese FH patients were enrolled after being diagnosed and treated at Department of Cardiology, The Second Xiangya Hospital of Central South University. Definition of FH was based on the standard (TC > 9 mmol/L and LDL-C > 5 mmol/L) formulated by European Society of Cardiology (ESC) and the European Atherosclerosis Society (EAS) (European Association for Cardiovascular Prevention & Rehabilitation et al. 2011; Goldberg et al. 2011; Hovingh et al. 2013). We have also taken CHD and xanthelasmas patients into account. Two hundred unrelated healthy Chinese subjects were recruited as control subjects to detect whether any sequence changes might be a common polymorphism (Xiang et al. 2014). Clinical data and detailed family history were collected for each subjects.

### Methods

#### DNA extraction

Genomic DNA was extracted from peripheral blood of all the subjects by using a DNeasy Blood & Tissue Kit (Qiagen, Valencia, CA) as previously described (Xiang et al. 2014).

#### Mutation sequencing

The entire coding regions and flanking intronic sequences of *LDLR* (NM_000527) and *PCSK9* (NM_174936) together with the p.R3527 mutation (part of exon 26) of *APOB* (NM_000384) were performed with polymerase chain reaction (PCR; primer sequences will be provided upon requests). Sanger sequencing was applied by the ABI 3100 Genetic Analyzer (ABI, Foster City, CA).

#### Multiple sequence alignments and bioinformatic prediction of mutation

The standard sequences of *LDLR*, *PCSK9* and *APOB* refer to Ensemble database. The polyphen2 (polymorphism phenotyping, http://genetics.bwh.harvard.edu/pph2/) (Sunyaev et al. 2000), Sorting Intolerant From Tolerant (SIFT, http://provean.jcvi.org/) (Ng and Henikoff 2003) and MutationTaster (www.mutationtaster.org) programs (Schwarz et al. 2010) will be used for the prediction of pathogenicity of genetic mutations.

## Results

### Clinic data

**Fig. 1:**
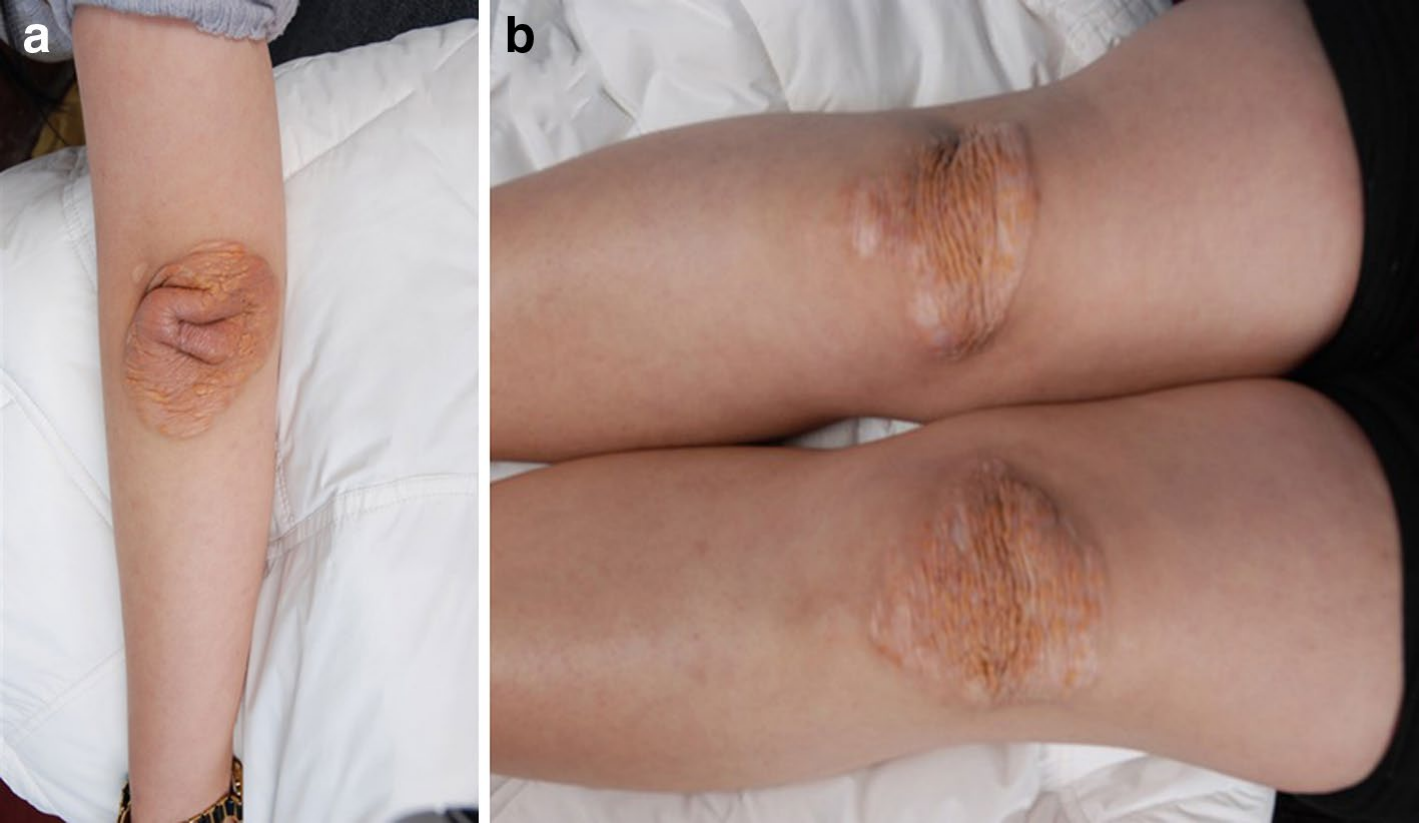
Xanthomas of FH homozygous individual (proband F3). On elbow (**a**) and knee (**b**)

A total of 11 unrelated FH probands were enrolled in this study, among whom four and seven showed homozygous and heterozygous phenotypes, respectively. Demographic details, clinical features, and lipid levels are shown in Table 1. In addition, the proband F3 had a history of xanthomas (Fig. 1), while proband F8 had a history of CHD.

**Table 1:** Characteristics and lipid levels of examined patients

Gender	Patient	Age (years)	TC (mmol/L)	TG (mmol/L)	HDL (mmol/L)	LDL-C (mmol/L)	Xanthoma	CHD
F	F1^a^	14	17.05	1.14	1.19	16.62	No	No
M	F2^b^	13	9.12	0.89	1.24	6.92	No	No
F	F3^c^	25	20.15	1.21	1.08	18.21	Yes	No
M	F4^b^	48	8.05	2.18	0.76	7.79	No	No
F	F5^b^	19	10.49	1.23	1.16	8.62	No	No
F	F6^b^	22	7.52	1.41	0.92	5.50	No	No
F	F7^b^	50	8.32	1.96	0.72	6.74	No	No
F	F8^c^	31	18.5	2.01	0.79	16.54	No	Yes
M	F9^b^	12	11.23	0.95	0.77	11.2	No	No
F	F10^b^	20	7.8	1.12	0.86	5.47	No	No
M	F11	7	18.91	1.03	0.94	16.84	No	No

In FH cases, TC and LDL levels are higher than 9 and 5 mmol/L

*M* male, *F* female

^a^Homozygous mutation, ^b^ heterozygous mutation, ^c^ compound heterozygous mutations

### Mutation spectrum

Eleven mutations in *LDLR* and one mutation in *APOB* were found by DNA direct sequencing in ten probands and co-segregated with all the affected members (Table 2). No mutation of *PCSK9* was found in any probands. Among these ten probands with variants, proband F1 carried the homozygous mutation, probands F3 and F8 carried compound heterozygous mutations. All three patients showed xanthomas, CHD or high TC and LDL-C levels. The mean serum TC was 18.57 mmol/l (min 17.05 mmol/l, max 20.15 mmol/l), and the mean serum LDL-C was 17.12 mmol/l (minimum 16.54 mmol/l, maximum 18.21 mmol/l). Other probands (F2, F4, F5, F6, F7 and F9) carried heterozygous mutations in *LDLR*. The mean serum TC was 9.12 mmol/l (min 7.52 mmol/l, max 11.23 mmol/l), and the mean serum LDL-C was 7.80 mmol/l (minimum 5.50 mmol/l, maximum 11.2 mmol/l). The proband F10 was detected a heterozygous mutation in *APOB*, whose serum TC was 7.8 mmol/l and serum LDL-C was 5.47 mmol/l. Currently none mutation of candidate genes was identified in proband F11. The serum TC was 18.91 mmol/l and serum LDL-C was 16.84 mmol/l.

**Table 2:** Mutations found in the Chinese and their predicted effect

Patient	Gene	Exon	cDNA	Protein	Protein prediction			PMID
					Mutation taster	Polyphen-2	SIFT	
F1^a^	*LDLR*	4	c.516C>A	p.D172E	Disease causing	Probably damaging	Deleterious	Novel
F2^b^	*LDLR*	12	c.1720C>A	p.R574S	Disease causing	Probably damaging	Deleterious	Novel
F3^c^	*LDLR*	5 9	c.760C>T/ c.1216C>A	p.Q254X/ No	Disease causing/ Disease causing	Unknown Unknown	Deleterious/Tolerated	Novel/ 17335829
F4^b^	*LDLR*	13	c.1954_1955delAT	p.M652GfsX16	Disease causing	Probably damaging	Deleterious	20538126
F5^b^	*LDLR*	4	c.682G>T	p.E228X	Disease causing	Unknown	Unknown	1301956
F6^b^	*LDLR*	4	c.485C>T	p.P162L	Disease causing	Probably damaging	Deleterious	12436241
F7^b^	*LDLR*	13	c.1897C>T	p.R633C	Disease causing	Probably damaging	Deleterious	9259195
F8^c^	*LDLR*	8	c.1132C>T	p. Q378X	Disease causing	Unknown	Unknown	11005141
		10	c.1448G>A	p.W483X	Disease causing	Unknown	Unknown	11810272
F9^b^	*LDLR*	12	c.1747C>T	p.H583Y	Disease causing	Probably damaging	Deleterious	7903864
F10^b^	*APOB*	26	c.10579C>T	p.R3527W	Disease causing	Probably damaging	Deleterious	7903864

^a^Homozygous mutation, ^b^ heterozygous mutation, ^c^ compound heterozygous mutations

### Novel mutations

**Fig. 2:**
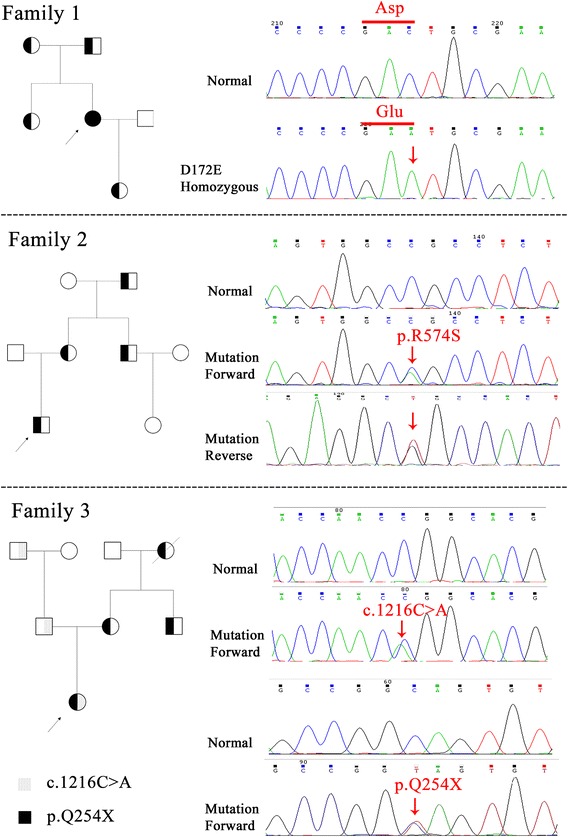
Pedigrees and sequencing results of the LDLR mutations of the families affected with FH. The hypercholesterolemic patient is indicated by a *black symbol*. The normal cholesterolemic individuals are indicated by *open symbols*. *N* normal, *M* mutant, *arrow* the proband

**Fig. 3:**
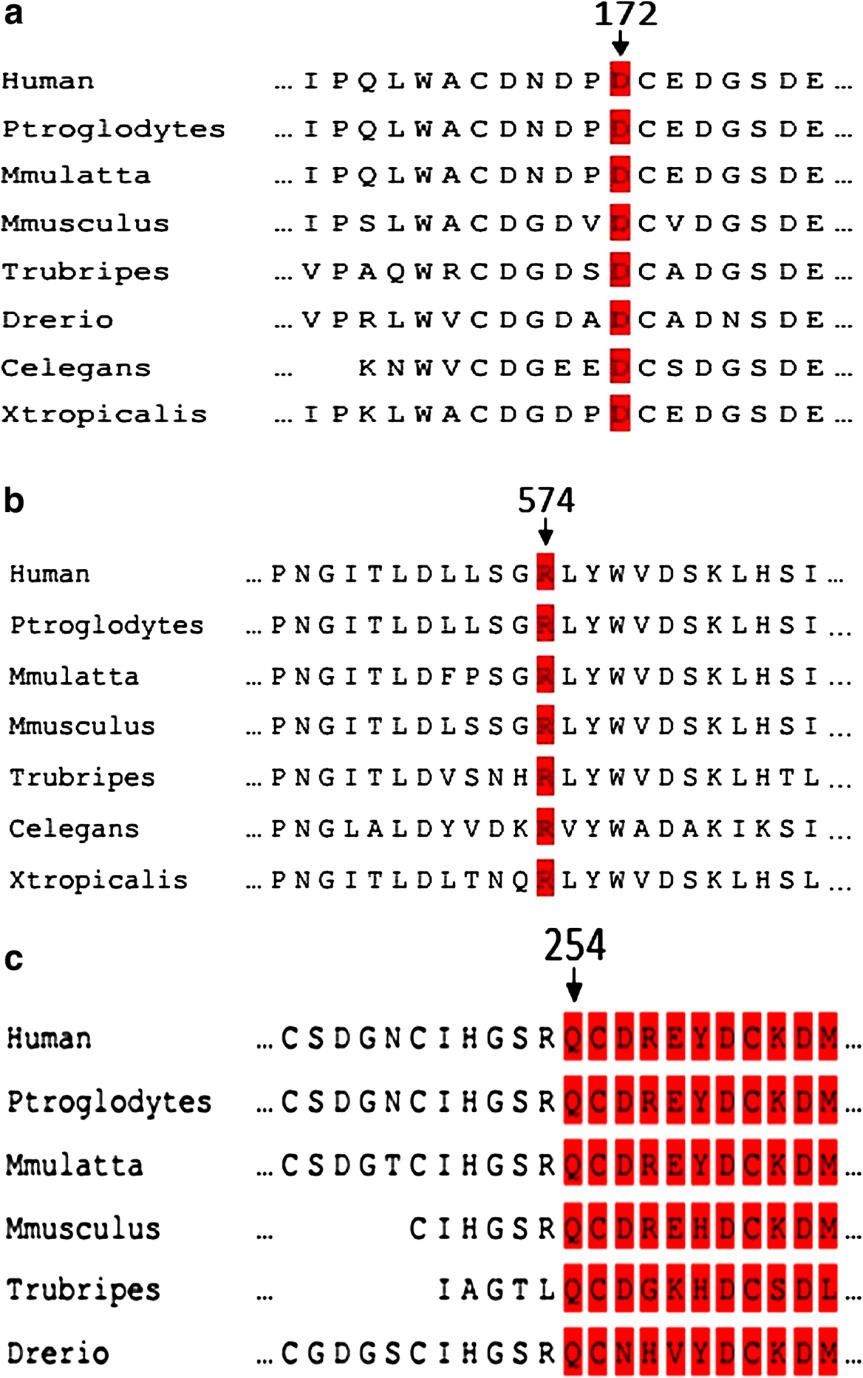
Analysis of the mutations of LDLR. Alignment of multiple LDLR protein sequences across species (from Ensemble). *Red columns* show conserved regions in site D172 (**a**), R574 (**b**) and Q264 (**c**) respectively

By sequencing analysis of *LDLR*, *PCSK9* and *APOB*, three novel mutations in *LDLR* (c.516C>A/p.D172E, c.1720C>A/p.R574S and c.760C>T/p.Q254X) were detected and co-segregated with the affected FH family members in our study (Fig. 2). These newly identified mutations were not found in either our control cohort of 200 patients, dbSNP or the Exome Variant Server database (http://evs.gs.washington.edu/EVS/). Alignment of LDLR amino acid sequences from Human, Ptroglodytes, Mmulatta, Mmusculus, Trubripes, Drerio etc., revealed that the affected amino acids were evolutionarily conserved (Fig. 3). Three programs for analyzing protein functions, MutationTaster, polyphen2 and SIFT, predicted that these three variants are disease causing, probably damaging and deleterious, respectively (Table 2). All three different algorithm based bioinformatics programs showed a consistent result of detrimental effect of these variants, suggesting that these three sites (D172, Q254 and R574) play important roles in the function of LDLR.

## Discussion and future perspective

According to EAS data, the estimated percentage of individuals diagnosed with FH in 2013 was less than 1% in approximately 180 countries/territories, including China. Moreover, China is a multi-racial nation, and such heterogeneous population is expected to harbor a number of novel gene mutations (Nordestgaard et al. 2013). In the present study, we employed direct sequencing to explore mutations of possible causative genes for FH. Twelve *LDLR* and *APOB* variants were detected, including three unique mutations (c.516C>A/p.D172E, c.1720C>A/p.R574S and c.760C>T/p.Q254X). The incidence rates of *LDLR* and *APOB* mutations were 82 and 9% in these Chinese FH families, respectively. These data corroborated previous reports demonstrating that over 85% of FH cases are due to hereditary mutations in *LDLR*, with the *APOB* variant (p.Arg3527) accounting for 5% of FH cases (Futema et al. 2014).

The novel mutations (c.516C>A/p.D172E, c.1720C>A/p.R574S and c.760C>T/p.Q254X) were detected in Families F1, F2 and F3, respectively. In Family F1, one homozygous and four heterozygous (c.516C>A/p.D172E) patients were identified. This mutation is found in the highly conserved ligand binding domain of LDLR, and may affect LDL binding (Gent and Braakman 2004). In Family F2, four patients (c.1720C>A/p.R574S) were diagnosed as FH. The substitution of the alkaline amino acid (Arg) by the polar but not charged amino acid (Ser) at position 574 of LDLR may be the genetic basis for FH. Proband F3 was a compound heterozygous mutation (c.760C>T/p.Q254X/c.1216C>A) carrier. The disease-causing SNP (c.1216C>A) is a splicing site that was used to exclude the natural splicing site, and causes a deletion of 31 bp from the mRNA, probably introducing premature termination of four codons after R406 (Bourbon et al. 2007). If the mRNA carries a nonsense mutation (c.760C>T/p.Q254X), it will be degraded by nonsense mediated mRNA decay. The LDLR protein without the C-terminal domain will not be found in the cell membrane. Therefore, serum TC and LDL levels were consistent with homozygous mutation carriers, such as proband F1.

Furthermore, *APOB* mutation (c.10579C>T/p.R3527W) was detected in Family F10. This mutation could influence the conformation and structure of APOB in the binding domain. This may decrease LDL degradation and increase TC and LDL-C levels (Gaffney et al. 1995). Besides, *APOB* mutations often show a lighter phenotype than *LDLR* and *PCSK9* mutations in patients. Our clinical and molecular data also confirmed this viewpoint.

Among all *LDLR* mutations, 27% (three out of eleven) of variants are found in exon 4. According to previous studies assessing Chinese FH patients, 24% of variants are found in exon 4 of *LDLR*, and our data are consistent with this percentage (Austin et al. 2004). Such a high frequency may be caused by the large exon size, but could be also related to selection bias.

In addition, no disease causing mutations in candidate genes were detected in proband F11, despite high TC and LDL-C levels in the patient. This might be caused by variations in other genes such as *APOC3* and *PNPLA5* (Jorgensen et al. 2014, Lange et al. 2014). Furthermore, CNVs also play a crucial role in FH for unique cases (Myocardial Infarction Genetics, Kathiresan, et al. 2009, Costelloe et al. 2012). Considering the serious phenotype of proband F11, we believe that genetic factors may have had a dominant effect. This will be identified through whole-exome sequencing in the future.

In conclusion, we detected mutations of *LDLR*, *APOB* and *PCSK9* in 11 Chinese FH families, among which ten were found to be deleterious mutations. Meanwhile, three novel *LDLR* mutations (c.516C>A/p.D172E, c.1720C>A/p.R574S and c.760C>T/p.Q254X) were identified. More patients were not available for statistical analyses, and no percentage of Chinese FH patients with positive genetic diagnosis could be revealed in this study. However, the present identification of three novel mutations and other mutations not only further supports the significant role of LDLR in FH, but also expands the spectrum of *LDLR* mutations. These new insights will contribute to the genetic diagnosis and counseling of FH patients.
